# Editorial to the Special Issue “Clinical Immunology in Italy, with Special Emphasis to Primary and Acquired Immunodeficiencies: A Commemorative Issue in Honor of Prof. Fernando Aiuti”

**DOI:** 10.3390/biomedicines11123191

**Published:** 2023-11-30

**Authors:** Alessandro Aiuti, Raffaele D’Amelio, Isabella Quinti, Paolo Rossi

**Affiliations:** 1 Faculty of Medicine and Surgery, Vita-Salute S. Raffaele University, 20132 Milan, Italy; 2 San Raffaele Telethon Institute for Gene Therapy (SR-Tiget), IRCCS San Raffaele Scientific Institute, 20132 Milan, Italy; 3 Department of Clinical and Molecular Medicine, Sapienza University of Rome, S. Andrea University Hospital, 00189 Rome, Italy; raffaele.damelio@gmail.com; 4 Department of Molecular Medicine, Sapienza University of Rome, 00185 Rome, Italy; 5 Research Unit of Clinical Immunology and Vaccinology, Bambino Gesù Children’s Hospital, 00165 Rome, Italy; rossipa@uniroma2.it; 6 Department of Systems Medicine, University of Rome “Tor Vergata”, 00133 Rome, Italy

Fernando Aiuti (Figure 1), born in Urbino on 8 June 1935, suddenly died on 9 January 2019, leaving a great void not only among his family members and those who knew him and appreciated his great humanity and acute intelligence, but in the entire immunological scientific community. We decided, therefore, to dedicate a Special Issue to the memory of Prof. Fernando Aiuti to highlight the great man and scientist that he was.

Fernando Aiuti was an internationally renowned immunologist, an outstanding, brilliant, and intuitive researcher with a solid post-doc scientific and clinical background in infectious diseases, who provided a relevant contribution to the birth of clinical immunology in Italy in the recent decades of the twentieth century. He was called to hold one of the first three chairs of clinical immunology in Italy in 1980 as full Professor of Clinical Immunology and Allergy at Sapienza, University of Rome, a role he held for over a quarter of a century up to 2007 (in the last ten years, he switched to the title of full Professor of Internal Medicine). In 1989, he organized the School of Specialization in Allergy and Clinical Immunology of Sapienza, University of Rome, which was directed by him from 1989 to 2007, training tens of young immunologists. His scientific fields of interest ranged from basic immunology, where he provided relevant contributions while working together with Hans Wigzell in Sweden in the early 1970s, through the setting up of a method for the identification of human T lymphocytes in normal subjects and immunodeficiency states [1,2,3], to clinical immunology, particularly in primary [4,5,6,7,8,9,10,11,12,13,14,15,16,17,18,19,20,21,22,23,24,25,26,27,28,29,30,31,32,33,34,35,36,37,38,39,40,41,42,43,44,45,46,47,48,49,50,51,52,53,54,55,56,57,58,59,60,61,62,63,64,65,66,67,68,69,70,71] and acquired [72,73,74,75,76,77,78,79,80,81,82,83,84,85,86,87,88,89,90,91,92,93,94,95,96,97,98,99,100,101,102,103,104,105,106,107,108,109,110,111,112,113,114,115,116,117,118,119,120,121,122,123,124,125,126,127,128,129,130,131,132,133,134,135,136,137,138,139,140,141,142,143,144,145,146,147,148,149,150,151,152,153,154,155,156,157,158,159,160,161,162,163,164,165,166,167,168,169,170,171,172,173,174,175,176,177,178,179,180,181,182,183,184,185,186,187,188] immunodeficiencies. The method that Aiuti and Wigzell set up to identify T lymphocytes was simple but at the same time brilliant, considering the period just a few years after the discovery of T and B lymphocytes [189], and the difficulty in handling and recognizing lymphocytes (only one method for identifying T lymphocytes had been discovered [190] just a few months before their publication). They prepared an anti-human T serum by inoculating in rabbits pure T lymphocytes, taking advantage of the availability of the peripheral blood lymphocytes of a patient with Bruton type agammaglobulinemia, a pathological condition characterized by a lack of B lymphocytes, thus avoiding any need to extensively purify the antigen to be inoculated [1]. Collaboration with the most renowned international groups of immunologists and clinical immunologists in Europe and the USA was a distinctive feature during his whole research career, in a period during which this attitude was uncommon in Italy. He was an expert member of the Commission for the Classification of Primary Immunodeficiencies of the World Health Organization for over twenty years (from 1975 to 1997), and his original contributions to the study of primary immunodeficiencies were in their diagnosis and pathogenesis [4,5,6,12,13,14,19,20,21,22,23,24,27,28,29,30,31,32,33,37,39,40,41,44,47,48,49,51,52,53,57,59,60,61,62,63,65,66,67,68,71], through their phenotypical characterization, and in treatment [9,10,11,13,16,18,34,35,36,38,42,43,45,46], with the then-pioneering bone marrow, fetal liver, and thymus transplantations, but even with thymic factors and immunoglobulins, for cellular and humoral defects, respectively. The safety of immunoglobulins for intravenous use by preventing HCV transmission was an issue he deeply cared about and actively pursued [50,54,55,56,64]. Together with Prof. Luisa Businco, who later became full Professor of Pediatrics at Sapienza University of Rome, he founded and managed, with the crucial support of Prof. Giuseppe Luzi, currently retired, the first Registry for Primary Immunodeficiencies in Italy [191,192], which now continues its relevant activity as the network for Primary Immunodeficiencies in Italy (IPINET). Together with other distinguished European Immunologists, Prof. Aiuti founded the European Group for Immunodeficiencies, which has grown now and developed into the European Society for Immunodeficiencies, which is very active in the field. An account of the beginnings of the European Society for Immunodeficiencies has been reported in this Special Issue by Roberto Paganelli, another student of Fernando Aiuti [193]. 

Since 1981, with the first description of the acquired immunodeficiency syndrome (AIDS), the main study interest of Fernando Aiuti was addressed to this new syndrome, in which he and his group provided many interesting scientific contributions to the epidemiology [75,80,83,84,89,97,98,100,110,112,115], pathogenesis [75,101,103,104,106,109,134,147,149,160], immunology [72,73,74,76,77,78,79,81,82,85,86,87,88,92,93,94,95,96,107,113,114,116,117,119,120,121,122,124,125,126,127,128,129,130,131,132,133,137,138,139,142,158,165,175], diagnosis [104,111,144,150,179], prophylaxis [135,143,145,151], and therapy [105,108,136,140,141,148,152,153,154,155,156,157,159,161,162,163,164,166,167,168,169,170,171,172,173,174,176,177,178,180,181,182,183,188], without neglecting the clinical [90,99,102,123,146] and psychosocial [118] aspects. In this period of his scientific career, Prof. Aiuti strongly felt the need to not be secret as a researcher to make the solidity of scientific information available to the general population and to remove false beliefs that could lead to the discrimination of categories of society, thus taking a public, social, and, later on, even political role in addition to the academic one. This new position can be witnessed in some of the books he wrote in that period. In 1993, in collaboration with the journalist Carlo Gallucci, Fernando Aiuti published a book entitled “Nessuna Condanna (No Conviction)”, in which 10 years after the identification of the first case of AIDS in Italy, he described the stories of different AIDS patients; the title reflects his feeling in participating empathically in the suffering of patients and in helping them as a man of science through the scientific development, but without any moral judgment on the risk behaviors (drug addiction, homosexuality, sexual promiscuity) for HIV infection [194]. In the same period, he wrote the book “AIDS Sapere = Vivere (Knowing = Living)”, an informative book for helping people understand, and thus how to avoid, the disease, which was then still invariably lethal [195]. More recently, following his retirement from the university, he had the time to write another very appreciated informative book with his former collaborator Prof. Giuseppe Luzi, titled “Il Nostro Meraviglioso Sistema Immunitario (Our Wonderful Immune System)”, to bring clinical immunology closer to the general population, thus continuing his educational mission, which was the hallmark of his life, alongside research and the care of frail patients [196].

Prof. Aiuti was enthusiastic, moved by a sincere curiosity and the tireless willingness to look for an answer to several scientific and clinical questions for scientific development and in the interest of patients, towards whom he always maintained a very warm, human, and protective attitude. His contagious enthusiasm was a powerful spring for the scientific growth of his group, and with his collaborators, he was demanding but faithful in his friendship. Fernando Aiuti had a very authoritative and fascinating personality, thus many postgraduate students were attracted to his field of research, deciding to test their capacity in this field in a laboratory where the atmosphere was highly competitive at the international level; the feeling of the young collaborators was the awareness that they were facing relevant scientific problems by trying to construct new methods, through the innovative discipline of immunology, which was competitively managed in comparison to the excellent research groups in London, Paris, or New York, then among the reference sites of scientific innovation and high-quality research. Fernando Aiuti had many national and international collaborations; however, with some researchers, the relationship was even of sincere friendship. This was the case for Prof. Luigi Fontana, full Professor of Internal Medicine at the University of Rome, Tor Vergata, who had spent a period of study in Sweden for research in the same years as Fernando Aiuti, for the virologist Prof. Ferdinando Dianzani, full Professor of Medical Microbiology at Sapienza University of Rome and later on Dean of the Faculty of Medicine of the University “Campus Biomedico di Roma”, with whom Fernando Aiuti had a very fruitful collaboration on the study of human immunodeficiency virus (HIV), and for the pediatrician Prof. Luisa Businco, already mentioned above, with whom he described and treated many children with primary immunodeficiencies. All of them prematurely passed away. Among the collaborators of his laboratory, Prof. Maria Caterina Sirianni, who had worked for a certain period in Hans Wigzell’s laboratory in Sweden following the suggestion of Fernando Aiuti and was a great expert on natural killer cells and a hard worker, and Dr. Soccorsa Le Moli, a biologist who worked with enthusiasm in the laboratory of Fernando Aiuti, then in the laboratory of immunology organized by Raffaele D’Amelio in the Italian Air Force and spent a period abroad conducting research in the laboratory of Giampietro Corradin in Lausanne, Switzerland, very precociously passed away. The occasion of this Special Issue in honor of Fernando Aiuti even presents the opportunity to remember these researchers and recognize the crucial role they had in building up a solid and respected field of clinical immunology by sharing Fernando Aiuti’s scientific vision. 

Fernando Aiuti had many collaborators working in the Laboratory directed by him in his long scientific career, some of whom remained to work with him and made a scientific career in the University, the majority at Sapienza University of Rome, but even in other universities, as Franco Pandolfi, who became Professor of Internal Medicine at the Catholic University in Rome, or Roberto Paganelli at the University “Gabriele D’Annunzio” of Chieti-Pescara. The others continued their scientific careers in different hospitals, in the “Istituto Superiore di Sanità” or in the “Agenzia Italiana del Farmaco”, or even abroad, in the Netherlands, as Oscar Pontesilli. 

The contributions to this Special Issue represent a tribute that many researchers, in large part former students of Fernando Aiuti, wanted to witness, providing largely original articles, which would have been very appreciated by Fernando, who was an innovator, always engaged in new scientific projects. In fact, out of 14 papers published in the Special Issue, 10 are original observations, whereas the remaining 4 refer to reviews or introductions to this Special Issue.

Isabella Quinti started her scientific career soon after her graduation at the University of Rome in 1978 in the laboratory of Prof. Fernando Aiuti, spent a period of her life in the USA, at Harvard University in Boston, and is currently full Professor of Internal Medicine at Sapienza, University of Rome, and Director of the Regional Centre for Primary Immunodeficiencies at the Umberto I^st^ University Hospital, the same one which had been first organized and directed by Fernando Aiuti. Isabella Quinti and co-workers submitted an excellent paper, in which they demonstrated that COVID-19 did not increase the mortality of 471 adults with Severe Primary Antibody Deficiencies compared with the pre-COVID period. Moreover, anti-COVID-19 monoclonal antibodies did result in being protective, whereas the anti-COVID-19 vaccines did not [197]. This excellent study on a very large number of patients, considering the relative rarity of these diseases, is a relevant contribution precisely in the field of primary immunodeficiencies and follows the same strategy of Prof. Aiuti, that is, testing the best ways to protect vulnerable patients with primary immunodeficiencies.

Massimo Fiorilli, together with Marcella Visentini and other co-workers, published a very interesting paper on the opposite effect of COVID-19 vaccines on CD3^+^,CD4^+^,CD25^+^,CD127^low^ putative regulatory T cells, the mRNA vaccine being stimulatory and the adenovirus vectored vaccine being inhibitory, in 24 patients with hepatitis C virus (HCV)-positive cryoglobulinemic vasculitis and in 25 healthy subjects [198]. This is a preliminary pilot study; however, they demonstrated so clearly that a polarized opposite effect allows these innovative vaccines to be better interpreted in their mechanism of action. A similar result has been observed in patients with rheumatoid arthritis, vaccinated for influenza, thus suggesting that these post-vaccine kinetics of putative regulatory T cells in immune-mediated inflammatory diseases may act as a regulatory mechanism against possible vaccine-induced lymphocyte polyclonal activation [199]. Massimo Fiorilli started to work in Fernando Aiuti’s Laboratory already as a pre-graduate student and, as a full Professor of Internal Medicine, was later the successor of Fernando Aiuti in the direction of the chair of Internal Medicine, the School of Specialization in Allergy and Clinical Immunology, and the Regional Center for Primary Immunodeficiencies at Umberto I^st^ University Hospital and is currently retired. Marcella Visentini is a Professor of Internal Medicine and current Director of the School of Specialization in Allergy and Clinical Immunology, following the direction of Prof. Isabella Quinti. 

Gianpiero D’Offizi, who graduated in 1981 from Sapienza, University of Rome, and soon after started to work in Fernando Aiuti’s Laboratory and is now Director of a Division of Infectious Diseases—Hepatology at the National Institute of Infectious Diseases “Lazzaro Spallanzani” Hospital in Rome, demonstrated, with a series of co-workers in a beautiful publication, that the safety of a COVID-19 vaccine in 149 cirrhotic patients was not dissimilar from that of 149 healthcare workers, whereas immunogenicity was higher in cirrhotic patients. Male sex and previous HCV were associated with a lower anti-S immune response [200]. Thus, in these cirrhotic patients, the safety of an anti-COVID-19 mRNA high-dose has been confirmed, and immunogenicity has been surprisingly demonstrated to be higher than in healthcare workers.

Paolo Rossi and Paolo Palma, with a series of co-workers, published a very interesting and useful paper on the best methodological conditions to explore HIV reactivation by either infected peripheral blood mononuclear cells or isolated CD4^+^ T lymphocytes, stimulated in vitro with different activator molecules [201]. They could demonstrate that autologous plasma is preferably to be used in quantitative tests, whereas RPMI seems to be preferable in qualitative tests. Prof. Paolo Rossi is currently a full Professor of Pediatrics at the University of Rome, Tor Vergata, and Director of the Pediatrics Department at Bambino Gesù Pediatric Hospital in Rome. Even before his graduation he had started to work with Prof. Luisa Businco, who was a Professor of Pediatrics, and Prof. Fernando Aiuti. In the period 1983–1987 he was abroad, at the Laboratory of Tumor Cell Biology in Bethesda, and at Karolinska Institutet in Stockholm, and in that period he facilitated the collaboration between Fernando Aiuti and Robert Gallo by obtaining from the latter a cellular line infected with HIV, by which the serological diagnosis of HIV infection was made possible, in the first period following the discovery of HIV as the etiologic agent of AIDS, in the absence, therefore, of commercial diagnostic kits.

Enrico Scala and Roberto Paganelli, together with some co-workers, wrote a very interesting original brief report on the relevant role of the proteomic analysis, as a complement to the IgE sensitization study, in patients with deregulated IgE [202]. Moreover, by describing four cases of different types of hyper-IgE syndrome, they report the first case of hyper-IgE syndrome associated with X chromosome microduplication. Enrico Scala worked as a post-graduate in Fernando Aiuti’s laboratory for some years, is currently responsible for the Clinical and Laboratory Molecular Allergy Unit of the “Istituto Dermopatico dell’Immacolata” in Rome, and is qualified to teach Internal Medicine. Roberto Paganelli, whose detailed presentation and relationship with Fernando Aiuti is reported in an Editorial of this Special Issue [193], was a full Professor of Internal Medicine at the University “Gabriele D’Annunzio” of Chieti-Pescara and is currently teaching at UniCamillus in Rome. Enrico Scala and Roberto Paganelli, with Massimo Fiorilli and some other co-workers, published in 1998 a very interesting paper for better defining the function of the lymphocyte activation gene-3 (LAG-3) [203], a CD4-related, activation-induced cell surface molecule which has been recently identified as an immune checkpoint, thus a possible target of immune checkpoint inhibitors in cancer treatment. 

Roberto Nisini took his first degree in Medicine in 1983 under the guidance of Prof. Aiuti, and then he worked as a researcher in the Italian Air Force Laboratory of Immunology organized by Raffaele D’Amelio, another student of Prof. Aiuti. He spent some time abroad in different periods, in the USA, at the National Institute of Health, Cancer Institute, in Bethesda, and at Basel Institute for Immunology and at Basel University Hospital, Centre for Biomedicine, in Switzerland. He is currently Research Director at the “Istituto Superiore di Sanità” as a Chief of the Laboratory of Immunology in the Department of Infectious Diseases. Roberto Nisini is an excellent immunologist, and in this Special Issue, he has provided a very interesting paper, together with many co-authors, including Raffaele D’Amelio, on the production of new monoclonal antibodies specific for different epitopes of the spike protein of SARS-CoV-2 and its major variants, which may represent important additional diagnostic tools for COVID-19 [204].

Andrea Picchianti Diamanti is a researcher in Rheumatology at Sapienza, University of Rome. He specialized in rheumatology under the guidance of Raffaele D’Amelio, who was then a full Professor of Internal Medicine and is currently retired, and, after the specialization and PhD in Rheumatology, he continued to work at the Sapienza University with Raffaele D’Amelio, who is, after Massimo Fiorilli, the oldest student of Prof. Fernando Aiuti, having started to work in his Laboratory in 1972, one year after his graduation. Andrea Picchianti Diamanti, with a series of co-authors, including Raffaele D’Amelio, Bruno Laganà, Professor of Internal Medicine, who worked with Fernando Aiuti for a short period and with Raffaele D’Amelio for nearly 15 years, Simonetta Salemi, who specialized with Fernando Aiuti and worked with Raffaele D’Amelio for nearly 15 years, and Roberta Di Rosa, who worked with Raffaele D’Amelio for nearly 15 years, provided an interesting paper which demonstrates that a third dose of anti-COVID-19 mRNA vaccine may safely be administered to patients with rheumatoid arthritis, considering that it does not significantly increase disease flares and adverse events [205]. Moreover, it was found that being vaccinated for influenza was inversely associated with the onset of adverse events after the second vaccine dose. The markedly decreased effectiveness after the third vaccine dose is a probable consequence of the spreading of the Omicron variant, which largely escapes immune recognition.

Vincenzo Puro and Delia Goletti, who work at the “Lazzaro Spallanzani” National Institute for Infectious Diseases, with which Fernando Aiuti collaborated during his research activity, together with a series of co-workers, presented a paper describing the breakthrough COVID-19 infections in vaccinated healthcare workers. Breakthrough infections were observed in 42% of vaccinated subjects, independently of the levels of neutralizing antibodies and specific cellular immune response, thus confirming the lack of usefulness of post-vaccine immune monitoring to obtain insights on the possible “immune protection”, at least with monovalent vaccines containing the original Wuhan viral strain [206], considering the increasing spread of the immune evasive Omicron variant of SARS-CoV-2, and likely associated with the waning of post-booster immune response.

Alessandro Aiuti, the first son of Fernando Aiuti and a full Professor of Pediatrics at the University S. Raffaele in Milan, is one of the greatest experts in the world of gene therapy for primary immunodeficiencies, particularly in severe combined immunodeficiency for the defect of adenosine deaminase (ADA-SCID), provided, together with Maria Pia Cicalese, a talented pediatric immunologist, Assistant Professor at University Vita Salute San Raffaele, Daniele Canarutto, a brilliant physician–scientist at San Raffaele Institute for gene therapy, and other coworkers, a very interesting paper on the outcome of BCG vaccination in 12 ADA-SCID patients [207]. It has to be underlined that, considering the low absolute frequency of ADA-SCID, having collected 12 cases, all coming from outside Italy, this has only been possible because of Alessandro Aiuti being the director of a referral center for gene therapy in patients with ADA-SCID. The important message of this paper is that the necessary TB prophylaxis in patients with ADA-SCID should be conducted only after enzyme replacement therapy in order to prevent a possible disseminated post-vaccine mycobacterial infection as a consequence of the SCID. Alessandro Aiuti recently wrote a book “The Unexpected Cure” together with the science populizer Annamaria Zaccheddu, describing how the deadly HIV that his father studied and fought, after genetic engineering, became a vehicle for gene therapy for genetic disease and cancer [208].

Roberto Paganelli was a student of Fernando Aiuti, whose detailed profile has been reported in the tribute to Robert Alan Good and Fernando Aiuti he wrote in this Special Issue [193]. He with some co-workers provided a very interesting report on a case of Good Syndrome, a severe primary immunodeficiency first described by Robert Alan Good in 1954, characterized by hypogammaglobulinemia with thymoma, recurrent bacterial, viral, fungal, and parasitic infections, as well as autoimmune manifestations [209]. This hypogammaglobulinemia has a worse clinical course and prognosis than other humoral defects because it is associated with thymoma, thus including abnormalities of lymphocytes of thymic origin, able to mediate cellular immunity, then configuring a sort of combined immunodeficiency. Moreover, in this paper, an excellent, analytical review of the literature regarding this rather rare immunodeficiency has also been provided.

Ivano Mezzaroma is currently a Professor of Infectious Diseases at Sapienza, University of Rome, who since his graduation worked with Fernando Aiuti, in particular on HIV/AIDS. Based on his deep experience in HIV developed under Fernando Aiuti’s guidance, he, together with two co-authors, provided an excellent review on the multifactorial interconnection of immune activation in HIV-1 infection [210]. The chronic immune activation induced by different causes is not reduced by specific anti-viral therapy, which may block viral replication, but it is unable to induce viral eradication and to arrest the chronic immune activation with inflammation, which favors the so called “inflammaging”, able to promote the diseases of the old age, such as neurocognitive impairment, cardiovascular diseases, diabetes and metabolic syndrome, inflammatory bowel disease, bone abnormalities, and non-HIV associated cancers.

Paolo Maria Matricardi graduated in Medicine under Fernando Aiuti’s guidance and worked in his Laboratory for some time, before becoming a medical officer in the Italian Air Force and joining the Laboratory of Immunology as a military researcher in Immunology and Allergy, which was organized by Raffaele D’Amelio. He is currently a senior scientist and private dozent at the Charité Berlin University. He provided a particularly interesting review on very low IgE producers [211], a neglected topic in the literature and a condition which may cover allergic rhinitis and may be associated with very low levels of the other Ig isotypes, recurrent airway infections, autoimmune diseases, and malignancies.

In conclusion, the enthusiastic and qualified response to the invitation to dedicate a Special Issue on biomedicines to what represented Fernando Aiuti in the landscape of clinical immunology in Italy and worldwide has shown the great vitality of his cultural legacy and attitude towards continuous innovation in research. Fernando would have been very satisfied with seeing this highly qualified response from his former collaborators.

## Figures and Tables

**Figure 1:**
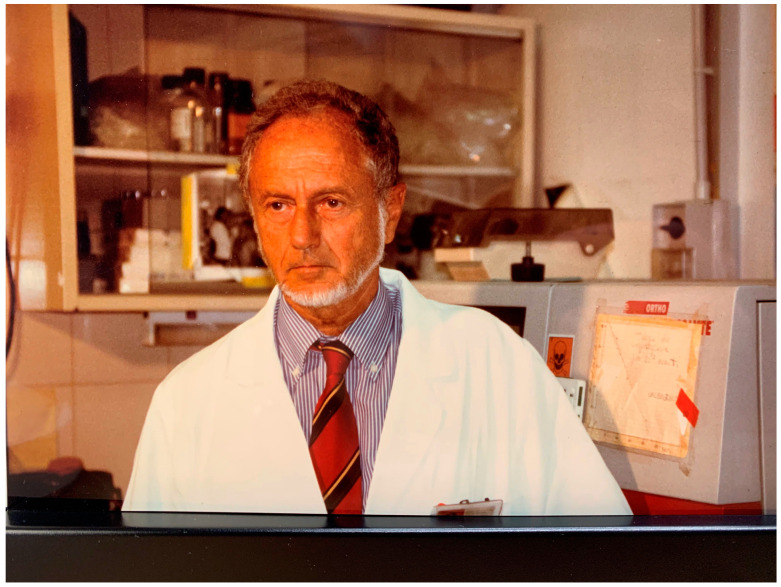
Fernando Aiuti in the Laboratory of Immunology of the University.
